# Why do people lift laboratory rats in different ways?

**DOI:** 10.1017/awf.2026.10101

**Published:** 2026-09-04

**Authors:** Jo Hockenhull, Trinity Camacho, Charlotte C. Burn

**Affiliations:** Pathobiology and Population Sciences, Royal Veterinary College, North Mymms AL9 7TA, UK

**Keywords:** Animal welfare, handling, human behaviour, qualitative research, *Rattus norvegicus*, Three Rs

## Abstract

Laboratory rats (*Rattus norvegicus*) are lifted using a wide range of methods that may differentially affect rat welfare and handler needs. Here, we analyse the free-text comments offered by laboratory rat handlers within an anonymous international questionnaire, to offer new insights into handlers’ reasons and concerns regarding how best to lift rats. This may help identify barriers to adopting potential best practice and elucidate what information is needed to develop future guidance on how to handle rats. A conventional qualitative content analysis approach was taken to analyse the data, and three key concepts were identified. The first was ‘a lack of consensus on what is best for the rat’, which highlighted a need to account for individual physical and behavioural differences between rats and between handlers, uncertainty about how to tell which methods are best for the rat, short- vs long-term implications, and lack of evidence. Secondly, ‘job-related influences’ included time pressure, influence of the procedures or purpose, and avoidance of being bitten by rats. Finally, a ‘strong anti-tail lifting sentiment’ was noted, with concerns about the effects of lifting rats by the tail in terms of rat welfare and research validity. The insights offered here suggest that, although the most harmful or impractical handling methods should be avoided, a range of different recommended handling methods may be needed. This would help safeguard rat welfare, feasibility and research validity whilst also accounting for individual differences between rats, handlers, and the varied purposes of the handling itself.

## Introduction

Laboratory rats (*Rattus norvegicus*) can be picked up using a wide range of different methods, which may vary in their effects on rat welfare and handler ease of use (Burn *et al.* 2023). Of particular concern is the use of lifting rats by the tail, which was first established to be highly aversive in mice (*Mus musculus*) by Hurst and West (2010). It is reportedly also associated with signs of stress in rats (Burn *et al.* 2023), although (O’Malley *et al.* 2026) observed no significant differences in behaviour or physiology between rats lifted by the tail, bodily or using a tunnel. The most common way to lift rats is a one-handed thoracic grip, shoulder saddle, which showed no significant reported improvements over tail lifting, unlike some of the less commonly used methods that were associated with fewer stress responses (Burn *et al.* 2023). There is therefore scope to refine rat handling to encourage non-aversive methods, and interventions with this purpose are ongoing both for mice (e.g. NC3Rs 2016) and more recently for rats (O’Malley *et al.* 2025). In a global pharmaceutical company, a policy change and training intervention reduced tail lifting from being used by 18% of respondents to just 2% after 2 years (O’Malley *et al.* 2025). However, implementing interventions becomes more challenging outside a single organisation that can adopt and ensure consistent policies, especially when successful implementation relies on changes in human behaviour.

The ideal lifting method would presumably be one that is comfortable and secure for the rat, and easy and quick for the handler, while preventing bites and scratches for the handler. However, the reasons why handlers use particular methods to lift rats have only recently begun to be explored. Understanding the barriers and motivators for using particular handling methods may be important for the success of any refinement interventions. Methods for lifting mice have been more widely researched (e.g. Hurst & West 2010; Clarkson *et al.* 2018; Gouveia & Hurst 2019; Henderson *et al.* 2020a). In an international survey of laboratory mouse handlers, respondents cited time constraints as a particular reason for lifting mice by the tail (Henderson *et al.* 2020b). Interviews of handlers of laboratory mice and rats in the USA and Canada suggested that, although most interviewees understood the benefits of low stress handling, the main barriers to switching from tail lifting were researcher attitudes, the perceived time needed to implement and use alternative techniques, and training personnel to use the techniques (O’Malley *et al.* 2022). Also, rat handlers in a multi-site pharmaceutical company reported that the main challenges in moving from tail lifting towards low stress handling methods (lifting bodily or within tunnels) were lack of ‘muscle memory’ and, again, perceived time taken to handle rats (O’Malley *et al.* 2025). The same study reported that tail lifting was mostly used for lifting ‘grumpy’ older rats.

The current study builds directly upon published quantitative data from an international survey of laboratory rat handlers (Burn *et al.* 2023) by analysing the free-text comments provided by respondents, offering fresh and potentially unexpected insights beyond those gained from the structured answer options. According to that survey, the most commonly used methods for lifting adult rats were shoulder saddle, chest and bottom support, and tail lifting, but at least ten different methods were used, plus ‘other’. Of particular relevance here, in a ‘tick all that apply’ question about the reasons why handlers may use their main method of lifting rats, handlers most commonly selected comfort of the rat, and the method being quick, easy, or how the handler had been trained to lift rats. Most respondents reported that they had no concerns about their chosen method, but a minority selected from a list of suggested concerns, particularly regarding discomfort for the rat, or themselves being bitten or scratched by the rat (Burn *et al.* 2023). The same survey revealed that laboratory staff were more likely to lift rats by the tail if taught by scientific researchers, whereas they used more supportive methods if taught on legal compliance training courses; and staff at USA laboratories were more likely to lift rats by the tail than those in the UK (Hockenhull *et al.* 2023). In the current study, we analysed the content of additional optional free-text data that some respondents voluntarily added following these structured questions. The main aim of this study was therefore to gain a deeper understanding about why staff use their chosen methods to lift laboratory rats.

## Materials and methods

### Questionnaire structure and content

Data were collected via a structured online questionnaire (Alchemer platform [formerly SurveyGizmo]; Alchemer®, Louisville, USA). The questionnaire contained questions on lifting and restraining guinea pigs (*Cavia porcellus*) and juvenile and adult rats (Burn *et al.* 2023). Only the section on lifting adult rats will be analysed here.

Respondents were asked to select their main method of lifting adult rats from the following options: base of tail with body support; tip of tail; cupping method; sandwich method; shoulder saddle; tunnel method; walk on method; bilateral sandwich method; chest and bottom support; and base of tail with no body support. This question and the questions that followed in the section on lifting adult rats were predominantly closed questions, but there were also five optional free-text boxes (Table 1). These five were each associated with separate questions relating to lifting rats (elaborations on the main handling methods; reasons why the method was chosen as their main handling method; any concerns about the method; whether the positives of the method outweigh the negative; how the rats behaved when handled). Additionally, the option was given to provide general free-text comments and suggestions for handling improvements at the end of the questionnaire (Table 1). It is the responses given to all these optional free-text questions that formed the dataset for this study.

**Table 1.** Questions associated with optional free-text boxes in a questionnaire for laboratory rat handlers about rat lifting methods. The numbers of respondents leaving free-text comments are also shown. Each respondent could leave any number of comments, so the total number of comments exceeds the 108 respondents who chose to leave comments. A total of 249 respondents answered the questionnaire about rat handling, but 141 left no optional comments. Table 1. long description.

Question stem	Free-text comments (n)
Which best describes the single main lifting method that YOU personally use for adult rats? (Q12)	19
Why do you use your main lifting method for adult rats? (Q13)	27
Do you have any concerns about your main lifting method for adult rats and, if so, what are they? (Q14)	24
Do you believe the positives outweigh the negatives of using this handling method, given the alternatives? (Q15)	62
What do some of the adult rats do when you lift them using your main method? (Q16)	21
Do you think handling practices for rats need to be improved in any way? (Q38)	70
Let us know if you have any further comments. (Q39)	21

The questionnaire was pilot tested by Royal Veterinary College (RVC) laboratory staff who work with rats, including an animal technician, an animal unit manager (also a NACWO and NTCO), a researcher, and a Named Veterinary Surgeon, and modified in response to the feedback received.

The questionnaire was live from 11th January–23rd February 2021. Recruitment was international, spanning all facets of laboratory animal use (for further details, see Burn *et al.* 2023).

### Ethical status

The study was reviewed and approved by the Royal Veterinary College’s Social Sciences Research Ethical Review Board (URN 2020 0283).

### Qualitative data analysis of free-text short answers

Questionnaire responses that included free-text answers to one or more of the seven optional questions (Table 1) were included in the analysis. Free-text responses were amalgamated and grouped by the main handling method reportedly used by the respondent. The three tail-lifting methods (base of tail with body support, tip of tail, and base of tail with no body support) were grouped together for analysis.

A conventional qualitative content analysis approach was taken to analyse the data (Hsieh & Shannon 2005). The process began with familiarisation and immersion in the data by one of the authors (JH), followed by a detailed reading to capture key thought or concepts through derived codes and noted impressions. After this initial analysis, JH and CB (who had also read every free-text comment) discussed the data and the codes that had been inductively generated by JH, ensuring consensus. The two authors then categorised the codes into meaningful groupings and each group was defined (Hsieh & Shannon 2005).

Only data from respondents who handled rats in a research laboratory setting at the time the questionnaire was completed or within the two years prior to completing the questionnaire were included in the analysis.

## Results

### Demographics

The questionnaire was completed by 249 respondents across 26 countries. Of these, 43% (n = 108) left comments in response to the optional free-text questions (Table 1) and so formed the sample for this study.

Within this sub-sample, respondents worked across 17 countries, with the most highly represented country being the UK (42%; 45/108). The majority of respondents worked in an academic research institution (65%; 70/108), with the pharmaceutical industry being the next best represented sector (18%; 20/108). The most common job role was animal technician (42%; 45/108), followed by scientist (17%; 19/108), animal unit manager (13%; 14/108), research animal veterinarian (12%; 13/108), husbandry and procedures advisor (8%; 9/108), ‘other’ (5%; 5/108), and research student (3%; 3/108).

**Figure 1.**
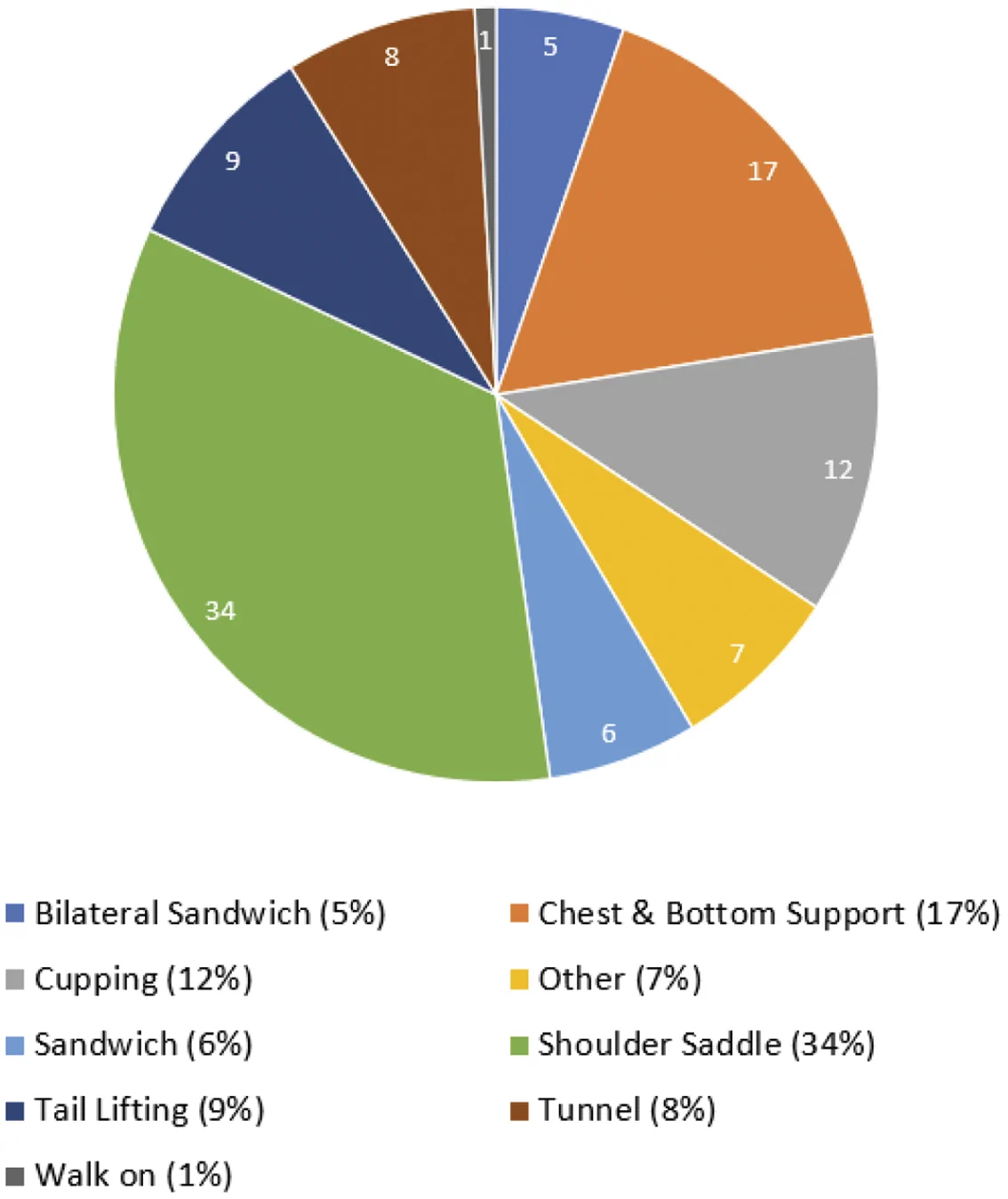
Pie chart of the main methods of lifting adult laboratory rats (*Rattus norvegicus*) used by questionnaire respondents who left free-text comments. The total number of the subset of respondents who both left free-text comments and answered the question about their main method of lifting adult rats was 108. The percentage of respondents using each method is shown on the chart and in the figure legend.

All adult rat lifting methods included in the questionnaire were represented in the sample (Figure 1).

### Results of qualitative evaluation of free-text short answers

Respondents’ comments were typically a single sentence; the longest comment was three sentences in length. Three key concepts were identified from the data: a lack of consensus on what is best for the rat; job-related influences; and a strong anti-tail lifting sentiment. These are described below supported by illustrative quotes, followed the main method used by the quoted respondent in brackets. Where necessary, quotes have been grammatically corrected for clarity.

### A lack of consensus on what is best for the rat

#### Differences between rats and between personnel

This concept encompassed the recognition that laboratory rats are not a homogenous group. The lifting method needed is likely to vary with the rats’ age, sex, size, experience (whether they are trained or habituated) and the procedure they are undergoing.

*“I think it is important to find the best way for the animal, because of this I would not go for one standard way to take a rat”* (Sandwich).

*“How I handle rats depends on how the rat wants to be handled – for excitable or fractious rats, I do things differently – shoulder saddle/v-hold; sometimes tunnel transfer, sometimes drape and cuddle”* (Chest and bottom support).

This lack of consensus was compounded by an interaction between the size of the respondents’ hands and the size of the rats, potentially with two-handed methods being best for lifting large rats using small hands vs one-handed methods for lifting small rats using large hands.

*“Pick up method in part depends on size of rat – I use saddle method if they are at the smaller end and not well handled – but have to move to two hands if bigger because I have small hands. If they are very familiar with me, I’d just scoop them up”* (Bilateral sandwich).

Even the physical height of the handler could influence the handling method.

*“I´m way too short to reach the back corners of our cages so I use the tube to extend my reach”* (Tunnel).

#### Uncertainty about which method is best for the rat

Some respondents explained that they found it challenging to identify the best method to lift rats and expressed uncertainty about whether their main handling method was the optimal choice for the rat.

*“I don’t believe it causes any additional stress but I’m unsure”* (Tail lifting).

Others reported that they based their decision on which lifting method to use on the absence of behaviours that could indicate aversion rather than the presence of more positive behaviours.

*“Most rats I have handled this way have not vocalised or became aggressive, which leads me to think this is an effective way of handling them without inducing stress”* (Sandwich).

“*Generally do not spend the time looking for the positive behaviours described so can’t say they wouldn’t happen”* (Shoulder saddle).

#### Conflict between perceived short- and long-term effects of lifting methods

Some respondents reported that their choice of lifting method was influenced by their perception of what was best for the rat outside of the moment of being lifted from the cage.

*“Restraint is stressful to the rats, and so I prefer a quick method to lift and get them out of the cage so I can start my examination without restraint if possible”* (Tail lifting).

*“A tube causes the rat to not be used to being physically touched causing issues later”* (Shoulder saddle).

The lack of consensus on the best lifting method reflects the diversity in the laboratory rat population, an apparent uncertainty regarding how to tell from the rat’s behaviour what is best for them, and the potential conflict between the short- and long-term implications of method choice, demonstrating the complexity of the issue. This was compounded by the lack of scientific evidence to inform decision-making, which formed a sub-category of this concept.

#### Lack of evidence to inform the use of different rat lifting methods

Respondents highlighted the dearth of research-based evidence to provide guidance on the best method to use to lift rats in a laboratory. A number of these comments made the comparison with the body of research that has been conducted on lifting laboratory mice which is much more extensive.

*“It would be useful to see work similar to that conducted in the mouse as to whether my current approach is the most optimal”* (Shoulder saddle).

*“There is less research on the tube method in rats as there is in mice. Basically, if the science can demonstrate the best practical way, that would be adopted for the majority of researchers”* (Bilateral sandwich).

The comment above implies that any advance in research into rat lifting would result in a change of handler practice. This is questionable, as illustrated by one respondent who was still using the method they were initially trained to use despite acknowledging that our understanding may have changed since then.

*“It is the way I was trained; over 2 decades ago. I’m sure the science/data has strengthened or weakened this. I’m not sure which”* (Shoulder saddle).

### Job-related influences

#### Time pressure

The length of time a lifting method takes in practice was reported to influence method choice. It was either the reason a method was chosen, *“Mainly because it is faster*” (Tail lifting), or the reason it was avoided “*Cupping is slower*” (Shoulder saddle).

Some respondents expressed the belief that there were kinder ways to lift adult rats, but these were not feasible given the lack of time they had available.

*“I think there are better ways of handling that are less stressful but many of these methods may take more time or have some other barriers”* (Bilateral sandwich).

*“If I had the time, I’d prefer to use more gentle methods. With the time I have, I feel it is the best option”* (Shoulder saddle).

#### Influence of the procedures or purpose

Rat-lifting method was associated with the role or purpose that the rat was used for. One respondent expressed that the procedure the rats underwent and the need for handler safety informed their choice of lifting method.

*“Animals are not in long enough to guarantee tameness before being infected with ACDP 3 agents, no risk at all can be taken to the handler even though I understand other methods are nicer for the animals. If we ever had clean animals of course other methods would be used*” (Tail lifting).

This distinction between ‘clean’ and ‘infected’ rats was mirrored by another respondent who had identified differences in the treatment of rats housed in the laboratory for research purposes as compared to those kept solely for training.

*“Our training rats love to be handled but we do have research rats that are housed without enrichment and not handled much. These animals are harder to handle”* (Bilateral sandwich).

The demands of the job that both the handler and the rats were in the laboratory to perform brought factors that influenced the choice of lifting method. While the handlers were predominantly influenced by a lack of time, either prior to rats undergoing a procedure or during their day to train or lift rats another way, the way the rats themselves were treated in terms of lifting and their daily management was influenced by the rats’ roles within the laboratory.

#### Self-preservation

The comments made by respondents on the concept of self-preservation were often follow-ups to other points they were making within the same free-text box, implying they were not the main focus of the respondent’s comment. However, choosing a lifting method based on the reduced risk of injury to themselves was understandably a common sentiment amongst respondents, and consequently formed a sub-category of the concept of job-related influences.

*“The risk of biting is low”* (Bilateral sandwich).

“*All of the other methods can cause the rat to bite, which I have been many times”* (Tail lifting).

*“However, if the rat is aggressive I will employ a different method to avoid biting [tail-picking]”* (Other).

It is interesting to note here that despite the strong anti-tail lifting positioning captured in the concept below, tail lifting was often the lifting method that respondents appeared to associate with reduced risk to themselves.

#### Strong ‘anti-tail lifting’ sentiment

The predominant lifting method that respondents made comments about, other than their own main lifting method, was tail lifting. These comments were negative across all respondents, but the reason for this varied. Some respondents explicitly compared their own choice of lifting method to tail lifting, with tail lifting always being a worse choice of method.

*“Definitely better than tail handling, but no evidence that it’s better than cupping etc”* (Shoulder saddle).

*“It’s a lot more humane for the rat, rather than tail handling”* (Shoulder saddle).

Other respondents cited their concerns about tail lifting as a method; for example the risk of injury it posed to the rat and the potential to impact the data collected. Similar concerns were not expressed about any other method.

*“Lifting rats by the tail should not be performed due to the additional risks of injury to the tail”* (Other).

*“A stressed rat by tail pick up would affect our results”* (Cupping).

Coupled with the sub-category above, this concept illustrates the conflicting views held by handlers around tail lifting. Despite the concerns associated with it, not only in terms of rat welfare but also the robustness of the data generated, it appeared that tail lifting was still seen by some respondents to have a place when lifting rats in a laboratory.

## Discussion

The findings shed new light on the reasons for using certain rat-handling methods, which could help inform future refinement initiatives. Analysis of these handler perspectives are relevant to culture of care discussions, and exemplify tensions between caring about rat welfare vs scientific or practical requirements (Davies *et al.* 2024). The results suggest that there is unlikely to be a one-size-fits-all solution, but instead that context may be important. There was a lack of consensus about the best method to use, partly because different methods could be best in different contexts and partly because evidence was lacking about which methods were best for rats. This may not be a barrier to refinement, if evidence suggests that there is more than one ‘best’ lifting method for rats and handlers. However, some respondents already felt strongly that tail lifting should be avoided to safeguard rat welfare or data quality.

### A lack of consensus on what is best for the rat

Despite ‘comfort of the rat’ having been one of the most commonly selected reasons for using a particular method to lift rats (Burn *et al.* 2023), the free-text comments sometimes seemed to express respondents’ difficulties in being asked to suggest just one main method for handling rats. For example, a new insight was that some methods may be difficult for staff to use if they have small hands, or if the rats they are handling are afraid of humans. Due to the brevity of the comments prompted within this survey, details of which exact methods suit different circumstances were often lacking. However, conceivably, a one-handed bodily grip (such as shoulder saddle) may be difficult for personnel to use if they have small hands and/or need to lift large rats. Tunnels may help solve this issue as long as they are sufficiently wide for large rats to enter and exit voluntarily, and as long as they are compatible with rats habituating to being handled by humans over the longer term. In one study, male rats seemed to develop aversion to entering tunnels as they grew larger, which the authors suggested could have been due to relative space restriction within the tunnel (Kylie *et al.* 2024). Any intervention to refine handling methods will need to consider issues such as these and may need to encourage use of more than one lifting method to ensure that rats can be handled well in a range of different contexts.

Another new insight from the free text comments was that some handlers reported using rat behaviour to help them decide which methods may be best for the rat, which suggests good practice and attention to detail. However, the focus seemed to be on observing stress responses, whereas there may be scope for also aiming to use handling methods that increase signs of positive welfare in the rats, indicating comfort and voluntary approach. Some measures of hand approach behaviour are already used in rat-tickling protocols, to help elucidate individual differences in how much rats enjoy the experience (Bombail *et al.* 2021) and could be extrapolated to handling contexts. We previously reported that respondents who lifted rats by the tail selected defaecation and struggling as common rat responses to handling significantly more than those using other methods, which could indicate rat stress (Burn *et al.* 2023). Conversely ‘sniffing the hand before lifting’ could indicate rats as having positive anticipation of handling, and that behaviour was selected least by handlers who lifted rats by the tail.

Comments also reflected a desire for evidence about which method(s) are most effective, either with regards to rat welfare or practicality for staff. In line with this, other comments suggested that respondents had not been able to, or had not attempted to, decide for themselves what seemed best for the rats based on the rats’ behaviour during handling. Rat handlers thus seem to want evidence-based guidance on the welfare impacts of different rat-lifting methods, similar to that available concerning mouse welfare (e.g. Hurst & West 2010; Clarkson *et al.* 2018). The first such evidence found no significant differences in rat physiology between those handled by the tail, body or tunnel (O’Malley *et al.* 2026), but further research is needed to help clarify this. With regards to practicality for staff, one respondent felt that if there was evidence suggesting the most practical method, that would be the method adopted by researchers. However, researchers are not necessarily any more likely than other personnel to change their own behaviour on the basis of evidence alone; for example, researchers were seemingly most likely to teach others to lift rats by the tail (Hockenhull *et al.* 2023), despite evidence that this is aversive in mice. Whilst some respondents used more than one method, the calls for evidence regarding practicality suggests that respondents were not confident about which they themselves would find most practical.

### Job-related influences

As found in previous studies (Henderson *et al.* 2020b; O’Malley *et al.* 2022; Burn *et al.* 2023), time pressure was a major influence on rat-lifting methods, with handlers who used tail lifting, but also shoulder saddle and bilateral sandwich, suggesting that they used these because they perceived them as quicker than some other methods. None of the free-text comments mentioned whether or not these perceptions were supported by data, such as those collected within respondents’ establishments. Published empirical data comparing durations between handling methods are lacking, but unpublished data summarised by NC3Rs (2016) suggested no statistically significant difference in timings between tunnel and tail handling of 98 mice.

Other aspects of the job that affected the chosen handling method included the tameness and infection status of the rats, which have been little reported as relevant factors before, and the need for handlers to avoid being bitten. The human-rat interaction may be important in these cases, because rats showed greater behavioural distress scores (including attempts to bite) when handled by humans who were less confident and who showed greater behavioural stress themselves when handling rats (Crichton Feddo *et al.* 2026). In our previous analyses, rat bites during handling were only reported by five respondents, each of whom used a different lifting method, but ‘rat bites and scratches’ were commonly selected as a concern by respondents who used two-handed lifting methods: sandwich, chest and bottom support and bilateral sandwich (Burn *et al.* 2023). The current analyses of free-text comments further showed that tail lifting was often stated as being used for rats that were difficult to handle and likely to bite, but several handlers instead used bilateral sandwich for such rats. Tunnel handling perhaps seems under-used in this context, because when used correctly (NC3Rs 2016) tunnels work via the instinctive escape response of a potentially fearful rat being to enter a refuge, and they also offer a hard barrier between the rat and the hand.

### Strong ‘anti-tail lifting’ sentiment

The voluntary comparisons that many respondents made between their main method of handling and tail lifting suggest that many of the respondents were familiar with the evidence of tail handling being aversive to mice and were extrapolating this to rats. Indeed, in our previous analyses of the quantitative answers to this survey, handlers who were aware of that evidence in mice were significantly less likely to use tail handling to lift rats than other lifting methods (Burn *et al.* 2023). Also, the respondents who mainly lifted rats by their tails had been significantly more likely to select ‘causing stress to the rat’ as a concern than were those using chest and body support. The current analyses of free-text reasons given for wanting to avoid lifting rats by their tails included risk of injury to the rat, and risk of unwanted effects on data quality caused by rat-handling stress. These reasons conflict with some of the above reasons given by respondents who used tail lifting as their main method of picking up rats, suggesting that handlers prioritise different perceived implications of handling.

### Study limitations

The free-text responses are likely to have been influenced by the preceding quantitative questions within the survey (Table 1) (Burn *et al.* 2023). However, the key concepts identified in the analysis spanned the data and introduced some original insights and subtle nuances, rather than directly reflecting respondent answers to a single free-text question, and some covered areas that were not raised in the questionnaire.

### Animal welfare implications

The comments offered by rat handlers in this study confirm that there is scope for refinement of rat-lifting methods, but that there is unlikely to be a one-size-fits-all solution. Instead, evidence is needed to establish a range of rat-handling methods that are comfortable and secure for rats and safe and feasible for handlers, and which can be deployed across a range of rats and handlers such that individual differences can be accommodated for.

## Long descriptions

### Table 1. Long description

The table consists of two columns: Question stem and Free-text comments (n).

* Which best describes the single main lifting method that YOU personally use for adult rats? (Q 1 2): 19 comments.

* Why do you use your main lifting method for adult rats? (Q 1 3): 27 comments.

* Do you have any concerns about your main lifting method for adult rats and, if so, what are they? (Q 1 4): 24 comments.

* Do you believe the positives outweigh the negatives of using this handling method, given the alternatives? (Q 1 5): 62 comments.

* What do some of the adult rats do when you lift them using your main method? (Q 1 6): 21 comments.

* Do you think handling practices for rats need to be improved in any way? (Q 3 8): 70 comments.

* Let us know if you have any further comments. (Q 3 9): 21 comments.

Navigate back to Table 1..
